# Skin Tone in Hyperspectral Imaging and Its Implications for Fairness in AI

**DOI:** 10.1002/jbio.70254

**Published:** 2026-03-31

**Authors:** Laurie S. van de Weerd, Nick J. van de Berg, L. Lucia Rijstenberg, Ralf L. O. van de Laar, Helena C. van Doorn, Heleen J. van Beekhuizen

**Affiliations:** ^1^ Department of Gynaecological Oncology Erasmus MC Cancer Institute, University Medical Center Rotterdam Rotterdam the Netherlands; ^2^ Department of Pathology Erasmus University Medical Center Rotterdam Rotterdam the Netherlands

**Keywords:** artificial intelligence (AI), health equity, optical imaging, skin pigmentation

## Abstract

Artificial intelligence (AI) is increasingly applied in healthcare, but concerns remain about bias affecting under‐represented groups. We investigated whether skin tone is systematically encoded in hyperspectral imaging data and how this affects classifications. Images were collected from 45 healthy women of the upper leg skin and vulvar mucosal tissue. Skin tones were grouped using the individual typology angle scale. Physiological parameters (oxygen saturation, haemoglobin, water and near‐infrared indices) were compared across groups. Unsupervised and supervised classification models were evaluated. Skin tone values ranged from −0.7 to 75.8 (20 very light, 9 light, 9 intermediate, 7 tan and 2 brown). All physiological parameters differed significantly across groups (*p* < 0.001). Unsupervised learning achieved 38.5% balanced accuracy, whereas supervised learning reached 71.4%, with high accuracies for tan (94.6%) and brown (95.0%) groups. Skin tone influences HSI data; it may act as a confounder in AI models, underscoring the need for diverse datasets to ensure equitable performance.

## Graphical abstract

This study investigates whether skin tone is systematically encoded in hyperspectral imaging (HSI) data and how this influences AI‐based classification. The results show differences in classification performance across skin tones when using both unsupervised and supervised learning methods, indicating the presence of potential bias.

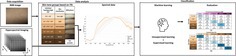

## 1 Introduction

The role of artificial intelligence (AI) in healthcare is rapidly expanding, with demonstrated value in cancer recognition, image reconstruction and treatment planning [1].

Nevertheless, its integration raises significant ethical and legal concerns, including data privacy, unclear accountability and algorithmic bias [2]. Furthermore, there is still a notable imbalance in the distribution of the benefits of AI, as data from several studies have shown that algorithms can perform poorly when applied to subpopulations [3]. A study by Chen et al. investigated the disparate impact that AI might have on intensive care unit (ICU) mortality [4]. They found that the model disproportionately focused on confounding health inequality factors, such as a person's race, gender and insurance type. Additionally, the model had a different error rate when predicting ICU death across groups. Because of this, the authors recommend checking for bias in AI models by comparing the accuracy of the predictions for different demographic groups.

Developing robust, unbiased and generalisable AI models requires diverse datasets [5]. Training a model involves the model learning to recognise and replicate patterns, features and styles from the data. There is a risk of bias being encoded in these models through non‐representative training samples or model evaluations based on narrow metrics [6]. Even seemingly technical parameters (e.g., for loss functions) can bring disadvantages to undersampled groups [7, 8]. This may mean that state‐of‐the‐art AI models can underperform for certain genders, ethnicities or insurance types [4, 9, 10, 11]. Yet, despite the manifold of alarm‐raising publications and the anticipated clinical impact of AI, research on practical implementations of algorithmic fairness in this field remains nascent [12].

### 1.1 Hyperspectral Imaging (HSI)

HSI is an emerging non‐invasive, radiation‐free, label‐free technique for scanning whole tissue areas at once [13]. An HSI camera captures a series of images and measures the intensity of the reflected light across different wavelengths, producing a reflectance spectrum for each pixel, which provides unique tissue fingerprints [14, 15]. Combining HSI with AI could be a valuable tool for tumour recognition [16, 17].

### 1.2 Application in Cancer Detection on Skin and Mucosa

AI for skin cancer detection is widely investigated [18, 19]. Vulvar cancer can arise from the skin or mucous membranes of the vulva, including the labia majora, labia minora, clitoris, vaginal opening and perineum [20]. Primary treatment involves surgery, in which the tumour is removed with histologically tumour‐free margins. However, general agreement about the optimal width of the resection margin is lacking. Removing too much healthy skin might lead to loss of function, while insufficient tissue removal can lead to a re‐resection. Accurately determining the extent of microscopic cancer cell spread remains challenging. A possible solution could be the use of HSI. Pachyn et al. studied HSI in free flap surgery, focusing on how skin tone affects HSI‐derived tissue indices [21]. They employed the Fitzpatrick skin tone classification and the individual typology angle (ITA) as an objective skin tone classification based on colour illumination parameters. Their results showed that pigmented skin impacts Hb‐related measurements, potentially altering tissue indices. Pigmented skin lesions, such as basal cell carcinomas, as studied by Räsänen et al., could complicate differentiation of malignant and non‐malignant tissue, particularly in patients with darker skin tones [22].

### 1.3 Goal

Our goal is to evaluate the extent to which HSI spectra have skin tone encoded and to determine the importance of collecting a diverse dataset for algorithm training. Despite growing awareness that bias in AI development can undermine equitable algorithm performance, follow‐up research and concrete strategies to mitigate this bias remain insufficient. In the context of tumour detection in the vulvar region, where the tumour can be located on the skin, the variability of skin tones must be carefully considered. In this study, we assembled HSI data from the upper leg skin and mucosal tissue of the vulva of healthy women with different skin tones to examine how skin tone differences might act as a confounder in AI models that rely on spectral data. We focused exclusively on the natural skin tone of healthy individuals to minimise variation from environmental factors, such as extensive sun exposure or underlying medical conditions. By controlling these factors, we aim to assess whether skin tone differences alone can be recognised from normalised spectral data and from physiological parameters derived from them, supporting the development of more equitable AI systems.

## 2 Materials and Methods

### 2.1 Participants

Participants were recruited at the Erasmus University Medical Center, Rotterdam. First, women aged 18 years and older who have no history of vulvar disease, no use of chemotherapy and no recent use of tanning beds were asked to participate in the study between July 2023 and October 2023. Additionally, a second call was used to obtain a more diverse dataset. Informed consent was obtained from all participants. The study was carried out according to the standards outlined in the Declaration of Helsinki. All procedures involving patients have been approved by the Medical Ethical Committee of Erasmus Medical Center Rotterdam in the Netherlands (trial protocol MEC‐2021‐0902).

### 2.2 Instrumentation

The HSI system used in this study was the TIVITA 2.0 (Diaspective Vision GmbH, Am Salzhaff, Germany). This camera has a spectral resolution of 5 nm across a wavelength range of 500 to 1000 nm. It captures the HSI data and an ultra‐high‐definition RGB (red, green and blue) image simultaneously from a similar view angle on two separate sensors. For more details, see Appendix A, “Instrumentation” section.

### 2.3 Data Acquisition

Women were asked to take place in a gynaecological examination chair. The camera was positioned orthogonal to the skin surface at a working distance of 50 cm, which was controlled by an embedded focus system containing red and orange light indicators. Three images were acquired: first, an image of the vulva was captured. Next, a picture was taken that primarily focused on visualising the mucosal tissue. Finally, an image of the skin of the upper thigh was taken.

### 2.4 Data Processing

All data analyses were performed in MATLAB (R2024a, MathWorks, Natick, MA, USA). Data processing involved image calibration, glare removal and noise filtering.

#### 2.4.1 Image Calibration

Image calibration corrected for non‐uniform illumination and pattern noise [23]. White and black reference images were collected before each acquisition using a Spectralon 99% reflectance standard (SRS‐99‐011, Labsphere Inc., North Sutton, New Hampshire, USA) and a thick piece of black paper obstructing all incoming light, respectively. In the HSI data, the white reference sample was segmented and the mean intensity across the region of interest (ROI) was computed for each wavelength. The calibrated image was then calculated using the following equation:

$$I_{\mathrm{cal}}\left(x,y,\lambda \right)=\frac{I_{\mathrm{raw}}\left(x,y,\lambda \right)-I_{\text{dark}}\left(x,y,\lambda \right)}{I_{\text{white}}\left(x,y,\lambda \right)-I_{\text{dark}}\left(x,y,\lambda \right)}$$

where $I_{\mathrm{cal}}\left(x,y,\lambda \right)$ denotes the calibrated image, $I_{\mathrm{raw}}\left(x,y,\lambda \right)$ the raw image, $I_{\text{dark}}\left(x,y,\lambda \right)$ the dark reference image, $I_{\text{white}}\left(x,y,\lambda \right)$. The white reference image, *x* and *y* represent the pixel coordinates, and λ the wavelength band.

#### 2.4.2 Glare Removal

For each wavelength, glare pixels were defined as those exceeding the mean intensity plus five times the standard deviation and were removed.

#### 2.4.3 Noise Filtering

Spectral noise was reduced using a second‐order low‐pass Butterworth filter with a cutoff frequency of 0.3. Additionally, normalisation of the spectra was applied, which makes sure that the data are scaled between 0 and 1 [24].

### 2.5 Skin Tone Classification

#### 2.5.1 Image Annotation and Registration

After image acquisition, skin and mucosa were annotated together with an experienced gynaecological oncologist in Photoshop (Adobe Inc., CA, USA). Green (HEX #0cf517) and blue (HEX #330df7) overlays were used to indicate skin and mucosa, respectively. ROIs were selected with a margin from visible tissue type boundaries to ensure a clean dataset.

For registration, the 70th spectral band of the HSI data cube was selected in MATLAB and exported along with the annotated RGB image. Two reference points were used to align the RGB to the HSI image via scaling, rotation and translation transformations. Once alignment was achieved, the resampled annotated ROIs were converted to binary masks and multiplied with the HSI data, enabling tissue‐specific spectral feature extraction.

#### 2.5.2 ITA

Skin colour was defined using the ITA scale by transforming RGB images to the CIELab or CIE L* a* b*, colour space. Hence, the ITA values were not a direct derivative of the HSI data. The ITA scale is a standard observer model aimed at presenting colours in a way that is independent of devices or lighting. It has three axes—L* (lightness), a* (red–green) and b* (yellow–blue). Positive and negative a* values represent red and green values, respectively, which correlate with erythema. Positive and negative b* values represent yellow and blue values, respectively, and are correlated with pigmentation or tanning. More details can be found in Appendix A, “Individual Typology Angle (ITA)” section. For subgroup comparisons of the skin, the ITA scale was divided into six topology levels (see Table 1). Additionally, to visualise the relationships between parameters derived from the calculated CIELab values and the ITA values, scatterplots are made. Also, this was done for mucosa data to investigate a possible relationship between skin tone and mucosal colour.

**TABLE 1:** The ITA scale allows skin colour classification into six groups, from very light to dark skin, adapted from [20]. The third column from the left shows RGB images of skin samples in the different skin groups. The fourth column from the left shows the distribution of participants across skin groups. Additionally, the lowest and highest ITA values within each group are shown together with the mean ITA value and standard deviation per group.

Individual typology angle (ITA°)	Skin classification	RGB picture	Number of participants	Lowest ITA value	Highest ITA value	Mean ITA ± SD
ITA° > 55°	Very light	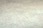	20	55.9	75.8	66.2 ± 6.8
41° < ITA° < 55°	Light	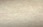	9	42.3	52.8	47.5 ± 3.1
28° < ITA° < 41°	Intermediate	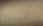	7	32.0	40.8	37.1 ± 3.2
10° < ITA° < 28°	Tan	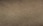	7	13.8	28.0	22.0 ± 4.6
−30° < ITA° < 10°	Brown	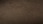	2	−0.7	7.5	3.4 ± 5.8
ITA° < −30°	Dark	Not available	—	—	—	—

#### 2.5.3 Spectral Data and Tissue Indices

To assess spectral differences that correspond to ITA classes, intensity spectra (mean ± SD) were visualised per class. Additionally, using a regression analysis, it was investigated whether the mean ITA values of skin and mucosal tissues correlate. Furthermore, physiologically descriptive features were derived from the HSI data. This included tissue water index (TWI), tissue haemoglobin index (THI), oxygen saturation (StO_2_) and the near‐infrared index (NIR‐index) (formulas can be found in Appendix A, “Spectral Data and Tissue Indices” section). All the tissue indices are given in arbitrary units (a.u.), ranging from 0 to 100, where 0 is equivalent to low parameter content and 100 to high parameter content. Therefore, these metrics do not present absolute amounts or fractions.

#### 2.5.4 Skin Tone Encoding in Unsupervised HSI Clustering and Supervised Learning

To assess potential bias in AI models, we evaluated whether skin‐tone diversity affects spectral cluster separability using a Gaussian mixture model (GMM). This model allows us to assess whether spectral features naturally separate into groups that correspond to different skin tone classes. First, a principal component analysis was performed on the spectral data to reduce the data dimensionality. The first 10 components were used, accounting for 98.4% of the variance in the data. After this, clusters were calculated with the GMM (the parameters of the GMM model can be found in Appendix A, “Skin Tone Encoding in Unsupervised and Supervised HSI Clustering” section).

Additionally, to assess whether HSI data encode skin tone information in a form usable by supervised models, we trained a fully connected network (FCN) to predict ITA skin tone classes. The FCN was trained on the normalised spectra, using an 80/20 train‐test split of the data. Given that the brown group consisted of only two participants, a twofold cross‐validation was performed. For training of the model, a subset of the data was used; that is, a random sample of 1000 data points was used per participant. The loss function used during training was a cross‐entropy loss function. The full network design can be found in Appendix A, “Skin Tone Encoding in Unsupervised and Supervised HSI Clustering” section. The full network design can be found in Appendix A, “Skin Tone Encoding in Unsupervised and Supervised HSI Clustering” section.

### 2.6 Statistical Analysis

Linear regression analysis was performed to examine the correlation between the measured colour of the skin and the mucosal tissue. Physiological parameters were analysed with a one‐way ANOVA and Tukey's Honestly Significant Difference (HSD) (*α* = 0.05) after confirming normality. Model performance (GMM and FCN) was evaluated using confusion matrices comparing the predicted labels and the actual labels. Prediction accuracy was calculated from these confusion matrices. In the case of the GMM, prediction accuracy was compared to the chance level, that is, exceeding the 20% accuracy expected from random assignment across five classes. Additionally, the Adjusted Rand Index (ARI) was computed between the predicted clusters and the ground truth labels to evaluate the clustering performance of the GMM.

## 3 Results

### 3.1 Participants

In total, 47 participants were included in the study. Measurements of two participants did not succeed due to technical issues. This resulted in a total of 45 participants for the skin analysis and 41 for the mucosa analysis (four lacked mucosal data). The age of the included participants ranged from 30 to 64 years (median: 53 years). The BMI ranged from 19.3 to 45.3 (median: 23.9). Of these participants, 24 were postmenopausal, of whom 1 was due to oophorectomy, 17 were premenopausal, 3 were perimenopausal and 1 participant had an intrauterine device (IUD), precluding menopausal status determination. All participant characteristics can be found in Appendix B.

### 3.2 Skin Tone Classification

The calculated ITA values and skin tone classes can be found in Table B1. In total, 20 participants had a very light skin tone, 9 had a light skin tone, 7 had an intermediate skin tone, 7 had a tan skin tone, 2 had a brown skin tone and none had a dark skin tone. The ITA values ranged from −0.7 to 75.8. Additionally, the mean ITA and the standard deviation (SD) were calculated per skin group; this can be found in Table 1.

#### 3.2.1 Spectra

**FIGURE 1:**
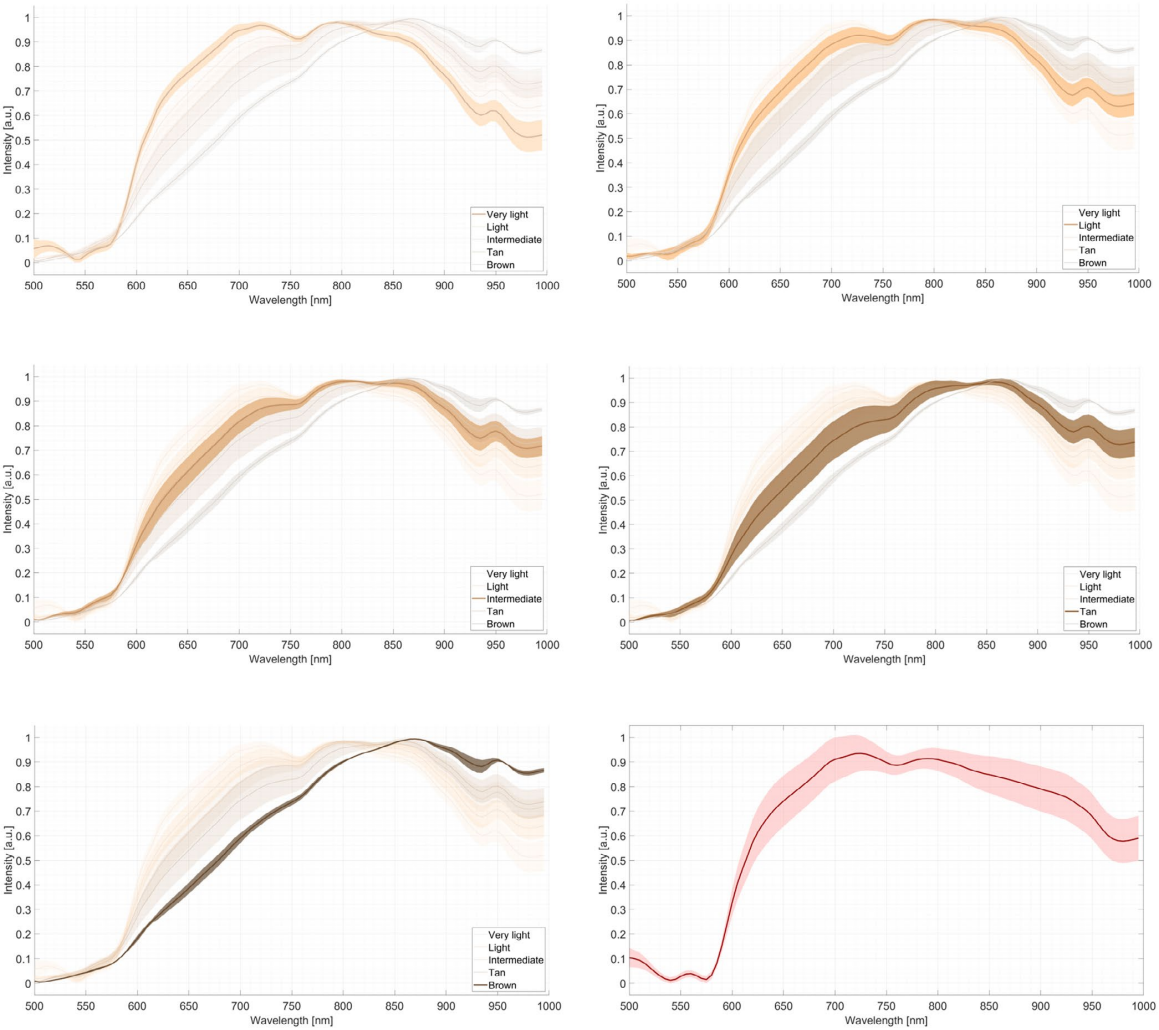
Intensity of the reflectance spectra per skin group (mean with standard deviation). The plot on the right side of the bottom row represents the data on mucosal tissue (mean with standard deviation).

The mean intensity of the skin groups, very light, light and intermediate, had a similar shape (see Figure 1). As reported by Kozlova et al. [25], haemoglobin exhibits absorption bands near 542 and 577 nm, while deoxyhaemoglobin absorbs near 555 nm, which can cause a ‘W’ shape in spectral curves [25]. Similar valleys are observed in our spectra, especially for lighter skin tone groups, with overall intensity decreasing as pigmentation increases.

The spectrum of the mucosa exhibits the most pronounced W‐shape. Also, a difference between 900 and 1000 nm can be seen between the skin and mucosal tissue. In this region, there are influences of water, lipid and protein absorption, plus scattering effects. The skin shows a more complex layered structure, whereas the mucosa is thinner and more homogeneous, showing smoother spectra.

#### 3.2.2 ITA Values

**FIGURE 2:**
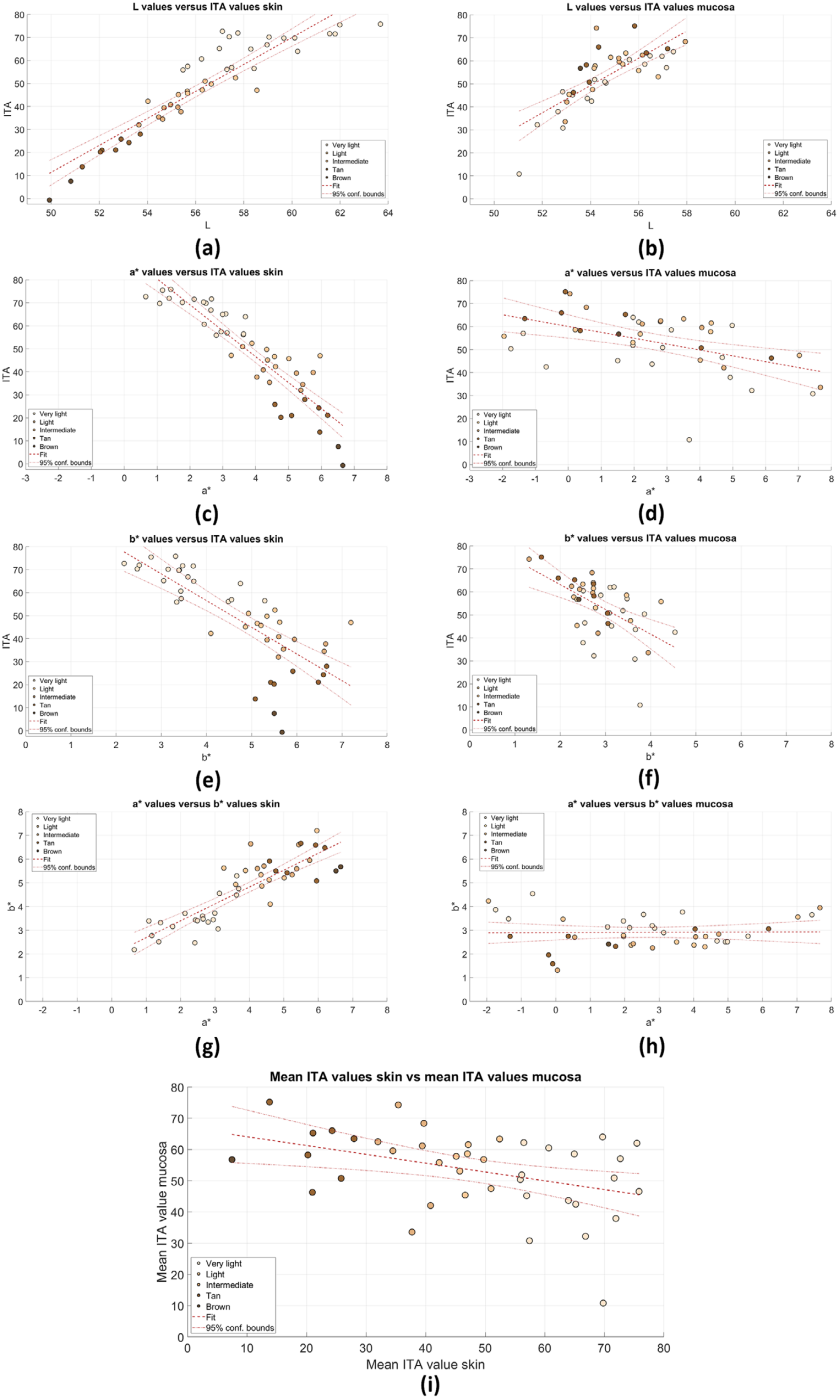
Scatterplots illustrating the relationships between colourimetric parameters derived from the calculated CIELAB values and the ITA values. The panels (a, c, e and g) show the *L* values versus the ITA values, the b* values versus the ITA values, the a* values versus the ITA values and the a* values versus the b* values, respectively, for the skin. The panels (b, d, f and h) show the *L* values versus the ITA values, the b* values versus the ITA values, the a* values versus the ITA values and the a* values versus the b* values, respectively, for the mucosa. The panel (i) illustrates the relationship between the SITA values of the skin and the ITA values of the mucosa.

**FIGURE C1:**
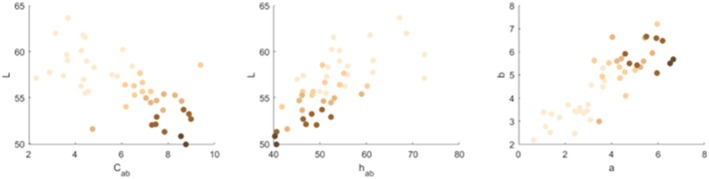
Scatterplots illustrating the relationships between colourimetric parameters derived from the calculated CIELAB values and the ITA values. (a) The *L* values versus the *C*_*ab*_ values, (b) demonstrates the *L* value versus the *h*_*ab*_ value. (c) The b* value versus the a* value. In all the plots, a linear fit is shown together with the 95% confidence interval.

Scatterplots illustrating the relationships between colourimetric parameters derived from the calculated CIELAB values and the ITA values are shown in Figure 2. Additional plots of colourimetric parameters can be found in Figure C1.

On the left side of the figure, all the scatterplots show the relationships for the skin. A positive correlation is observed between L* and ITA, which shows that the ITA is largely influenced by the lightness of the skin (correlation coefficient = 0.916). The a* and b* values show a negative correlation with the ITA values (correlation coefficients = −0.901 and −0.770, respectively), which suggests that the darker skin tones are associated with higher red and yellow components. A positive correlation is observed between the a* and b* values (correlation coefficient = 0.857); this implies that with increasing redness, the yellow components are also stronger.

On the right side of the figure, the relationships between the CIELAB values and the ITA values are shown for the mucosal tissue. A slightly positive correlation is observed between L* and ITA, which implies that the ITA value is slightly influenced by the lightness of the mucosa (correlation coefficient = 0.766). For both the a* and b* values, a negative correlation with the ITA values is shown (correlation coefficient = −0.503 and −0.574, respectively), which suggests that darker mucosal tissue is associated with higher red and yellow components. However, the correlation is less pronounced than in the case of skin tones. Finally, a slightly positive correlation was observed between the a* and b* values (correlation coefficient = 0.0133), which implies that with increasing redness of the mucosa, the yellow components also tend to become slightly more pronounced.

#### 3.2.3 Correlation Between Skin Tone and Mucosal Colour Tone

A negative correlation between the ITA values of the skin and mucosa was observed (see Figure 2). Regression analysis showed a significant relationship between the ITA values of the skin and mucosa (*p* = 0.0079). The root mean squared error (RMSE) was 11.7, which indicates moderate variability around the regression line. Although the regression model was statistically significant, the effect size was limited, as the indicated *R* ^2^ value was 0.167; that is, the ITA value of the skin is a significant but modest predictor of the ITA value of the mucosa.

#### 3.2.4 Comparison of Physiological Parameters Per ITA Class

**FIGURE 3:**
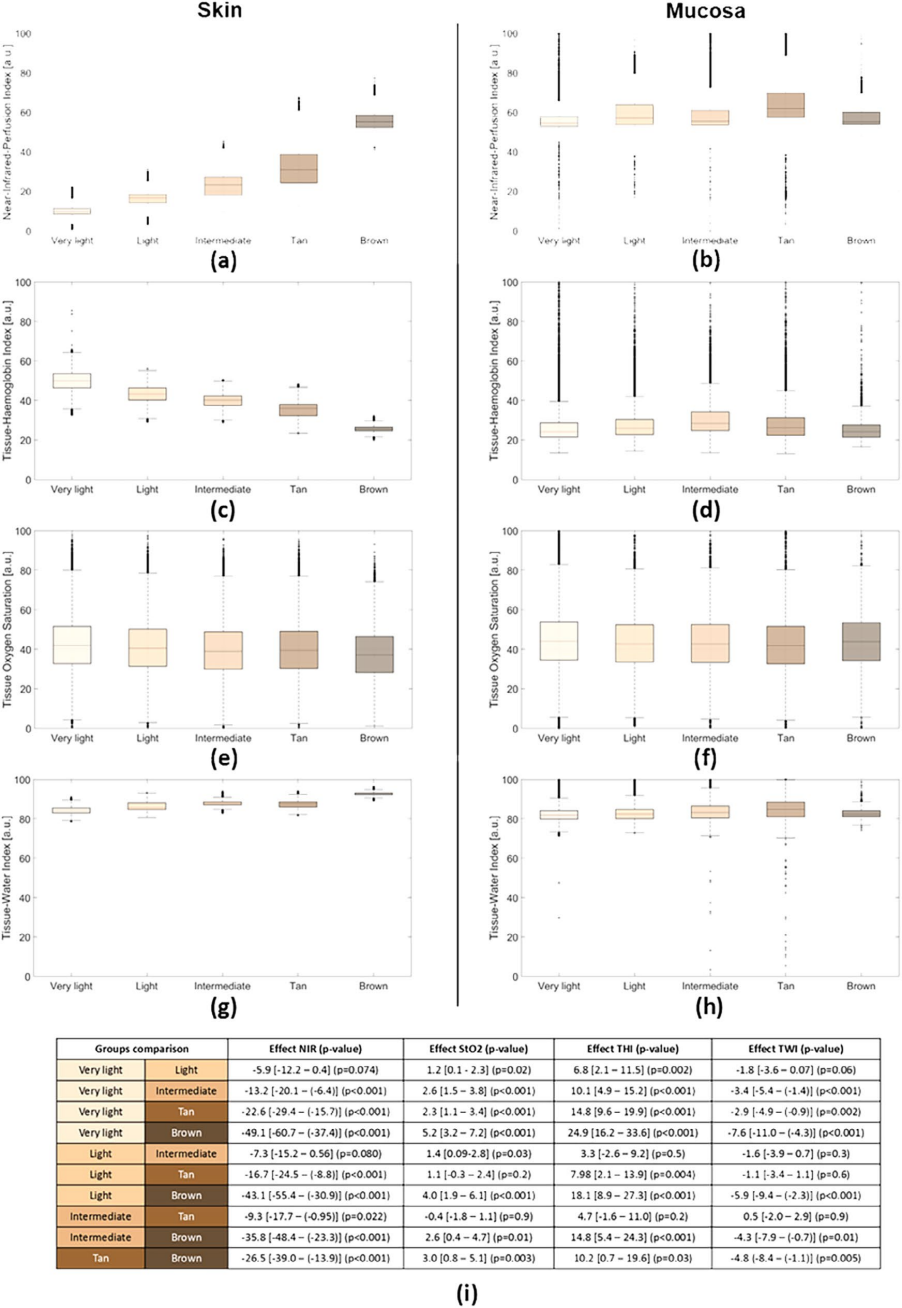
The physiological parameters: Near‐IR perfusion index, oxygen saturation, haemoglobin index and water index, derived from HSI data, are shown per ITA skin tone class on the left side (a, c, e, g). On the right side, similar physiological parameters derived for the mucosal tissue are shown (b, d, f, h). Additionally, the panel (j) shows a table with the effect sizes, the lower and upper bounds of the confidence intervals, together with the *p* values.

Boxplots of the distribution of the tissue indices regarding the ITA values are visualised in Figure 3.

The one‐way ANOVA showed that for all four physiological parameters, there was a difference between the ITA classes (*p* < 0.05). Post hoc Tukey HSD analysis showed which ITA classes differed significantly from each other. The effect size, lower and upper bounds of the confidence interval, and the *p* values for the comparisons between the physiological parameters of the different ITA classes can be found in Figure 3.

For the mucosal tissue, the one‐way ANOVA showed that there was a significant difference for the StO_2_ and the near‐infrared perfusion index between the ITA classes. Post hoc Tukey HSD analysis showed that there were no significant differences between the ITA classes.

#### 3.2.5 Skin Tone Encoding in Unsupervised HSI Clustering and Supervised Learning

**FIGURE 4:**
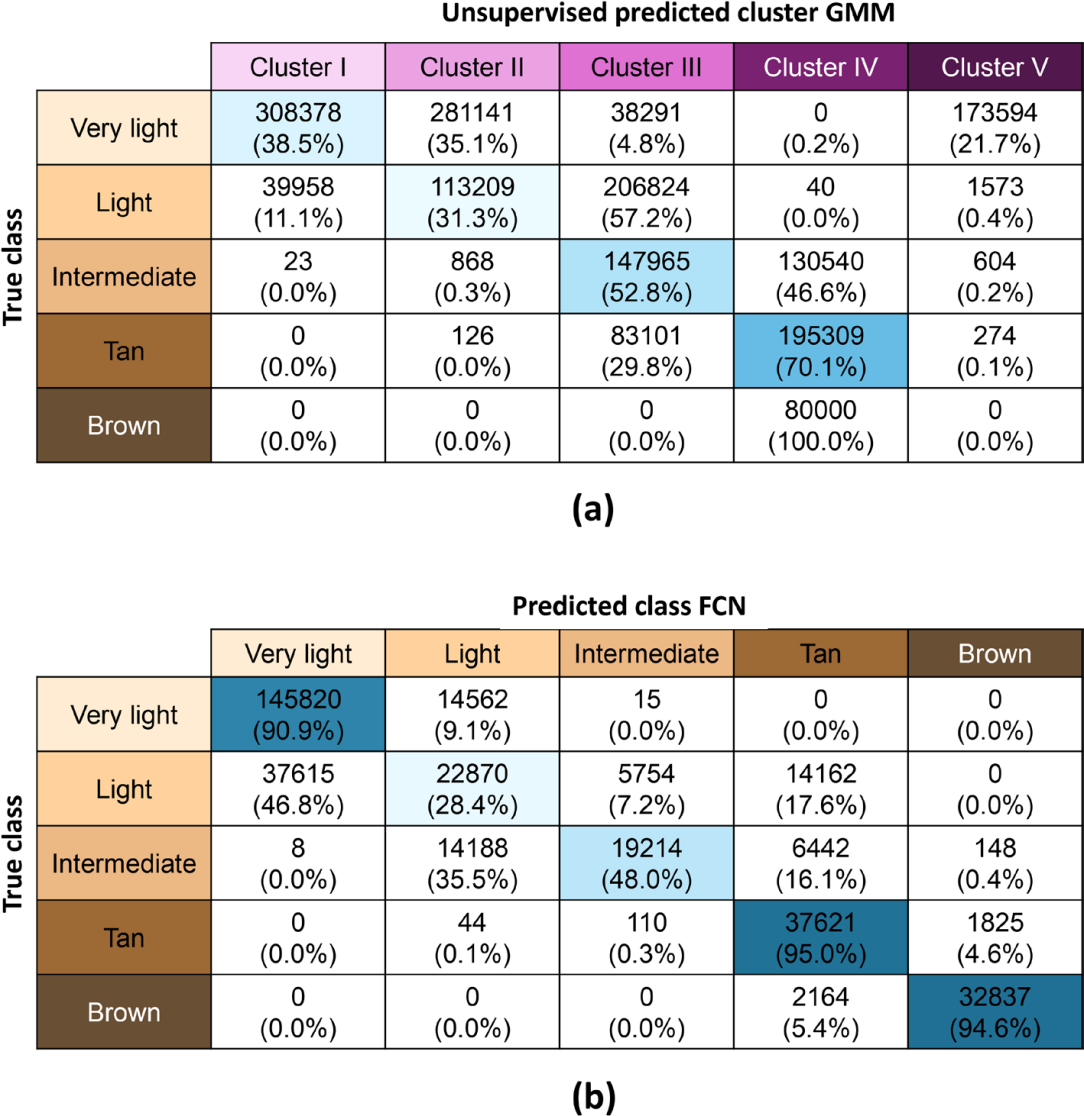
Confusion matrices (a) comparing the predicted clusters by the GMM model and the true classes and (b) comparing the predicted labels by the FCN and the true classes.

The correspondence of ITA and GMM clusters was evaluated using a confusion matrix; see Figure 4a. The accuracy of the correspondence between ITA classes and GMM clusters ranged from 0.0% to 70.1%. The overall balanced accuracy of the GMM was equal to 38.5%, substantially above the chance level (20%). The GMM achieved an ARI score of 0.35, indicating a moderate agreement between the predicted clusters and the ground truth labels. The results indicate that skin tone is encoded in the spectral data. The GMM grouped the ITA classes ‘tan’ and ‘brown’ together in one cluster and created an additional (fifth) cluster at the ‘very light’ skin tone level, shown in scatterplots according to the first two principal components in Appendix D.

**FIGURE D1:**
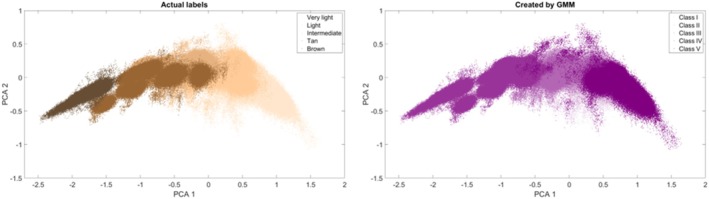
The first two principal components (PC1 and PC2) of the dataset are shown on the left, the actual ITA‐based labels, and on the right, the predicted skin type labels (GMM).

In Figure 4b, the confusion matrix of the FCN‐based ITA skin tone predictions is shown. A high classification accuracy is seen across three of the five ITA classes, including the ‘very light’, ‘tan’ and ‘brown’ groups, with an average accuracy of 93.5%; misclassifications were minimal and primarily occurred between the adjacent ITA classes. Overall, the balanced accuracy of the FCN was equal to 71.4%. For the ‘light’ and ‘intermediate’ groups, the accuracy was significantly lower. When looking at the scatterplots in Figure D1 also demonstrates that there is a lot of spectral overlap for the light and intermediate groups.

## 4 Discussion

Our study exposes a critical, yet under‐examined vulnerability in AI applied to HSI: the potential of skin‐tone‐related bias. Multiple analyses were performed to investigate the influence of skin tone on reflectance spectra, physiological tissue parameters and unsupervised and supervised tissue classification. These classifications evaluated the extent to which skin tone information is encoded in HSI data.

We observed that skin tones are consistently encoded in spectral data. However, it was not the sole dominant factor in cluster definitions, as the GMM identified an additional cluster within the very light skin tone region, likely based on spectral patterns unrelated to skin tone.

Complementing this, a supervised FCN predicted ITA classes from HSI data with 71.4% balanced accuracy, confirming that skin tone information is reliably present and extractable from the spectral signatures. These analyses were performed using normalised spectra; the findings indicate that skin tone information is not solely contained within intensity values but also in spectral shape, band ratios and wavelength‐dependent absorption features inherent to biological tissues.

Additionally, significant differences were observed in all four physiological parameters between ITA classes for skin, suggesting that the current explainable features may suffer from the same bias. These differences may arise both from genuine variations in tissue composition, such as melanin or haemoglobin content, but also from limitations in the equations used to derive these features, which often rely on simplified models. For mucosal tissue, no significant post hoc pairwise differences were found between the physiological parameters and ITA classes. Our results demonstrated that skin colour is a significant but modest predictor of mucosal colour. Additionally, the relationship between the colourimetric parameters derived from the calculated CIELAB values and the ITA values shows that mucosal colour and skin tone do not exhibit a clear association. Also, age and menopausal status are important determinants of mucosal characteristics, as the decline in oestrogen levels during menopause is known to affect the mucosa [26].

In our study, physiological parameters were computed using wavelength‐band ratios without accounting for differences in skin tone, a limitation also reported in other domains, such as reduced accuracy in pulse oximetry and wearable oxygen sensing in darker skin tones. Adjusting calibration procedures or modifying the formulas to account for skin‐tone variation could improve accuracy and reduce potential biases in HSI‐derived physiological measurements [27, 28].

Methods such as spectral unmixing, which separate individual components, could assist in the development of more interpretable features and clarify what is being measured and which factors most strongly influence the signal. For both simplified and more complex models containing physiologically descriptive features, validation across diverse populations is urgently needed. Clinically, this means that in future AI applications, such as skin cancer assessment, skin tone information should be included to ensure appropriate diagnostic use.

Despite promising results in HSI‐based detection of skin cancers, current literature lacks a systematic evaluation of model performance across skin tones. Recent work has shown that melanin‐related bias in HSI can distort perfusion indices, particularly in individuals with darker skin tones, highlighting the need for tone‐aware calibration in clinical imaging applications [21].

In dermatology, the Fitzpatrick skin type (FST) classification remains the gold standard for subjective skin tone assessment [29]. However, Pinchon et al. demonstrated that the FST has limited utility across several ethnic populations, including Asian, Arab and African–American groups, as its reliance on skin responses such as burning and tanning may be overly restrictive [30]. These limitations highlight the need for a more inclusive skin tone classification system. Therefore, objective colourimetric measures like the ITA may provide a more reliable and holistic characterisation of skin tone [31]. Pachyn et al. employed both the FST and the ITA scale to calculate tissue indices from HSI measurements and to assess the influence of skin tone on these measurements [21]. They reported that the two skin tone classifiers contain different information about the physiological state of a tissue. Moreover, they noted that FST is a body‐site‐independent characterisation that is primarily applicable to pigmented sites, whereas the ITA scale appears better suited for correcting skin‐tone‐related bias in tissue indices at specific body sites.

A limitation of our study is the reliance on ITA classes, which are tied to melanin‐based pigmentation, making it less applicable to mucosal tissues. Although ITA scores were calculated for mucosal tissue to establish a baseline, our analysis focused primarily on skin and mucosal tone variability was not explored in depth. In diseases such as vulvar cancer, in which both skin and mucosal tissue can be involved, the development of a new tissue tone scale (similar to ITA) for mucosal tissue may be required, possibly incorporating the CIELAB a* component. Finally, ITA scores were determined from RGB images. Annotation and registration of ROIs were performed manually in Adobe software together with clinicians. In future work, these steps could be automated to further standardise the processing pipeline.

A second limitation of our study is the relatively small cohort size. In total, 45 participants were included, of whom only 2 participants fell into ITA Class V and none in Class VI, limiting the generalisability of our findings. Nevertheless, there was a wide range of ages and different menopausal statuses among the participants. While this dataset was sufficient to identify potential bias risks in HSI‐based models, it is not yet adequate for developing a fair and robust algorithm for its intended use, that is, vulvar cancer detection. For instance, the outer skin‐tone groups achieved substantially higher performance, whereas intermediate skin tones (‘light’ and ‘intermediate’ groups) were misclassified more frequently due to considerable spectral overlap. This overlap is also reflected in their ITA scores, with a mean difference between these groups of only 10.4, compared to 15.1 or greater for other group pairs, indicating greater similarity in skin tone. In contrast, the ‘brown’ group contained only two subjects with low within‐group variability, which likely led to model overfitting. A broader and more diverse dataset is therefore required to better capture the subtle spectral variations within and between skin tone groups. Future work should prioritise expanding the dataset to cover a broader spectrum of skin tones and mucosal variations, also keeping in mind menopausal status.

We advise AI developers working with biomedical spectral data to adopt a structured, fairness‐aware approach to enhance equity in health. This includes (1) transparently reporting training set composition, particularly regarding demographic and phenotypic diversity, (2) actively striving to include a representative range of skin tones and tissue types in training datasets, (3) clearly defining and communicating the intended use and limitations of AI models and (4) implementing model auditing and fairness testing across subgroups. These steps should help in detecting unintended bias, realising equitable performance across diverse patient subgroups and ensuring reproducible and ethical deployment of AI algorithms.

To conclude, our results demonstrate differences in the classification performance of skin tones using unsupervised and supervised learning, which implies possible bias. By including physiological parameters into AI models, we aim to enhance the interpretability of the results and work towards creating more equitable models. We believe that understanding how skin tone can influence these models will help ensure that they perform equally well across all skin tones, ultimately leading to fairer and more inclusive AI systems.

## Conflicts of Interest

The authors declare no conflicts of interest.

## Data Availability

Research data are not shared.

## Appendix A

### Instrumentation

The camera employs a pushbroom scanning technique with a spatial resolution of 640 × 480 pixels and a field of view (FOV) of 8.0 × 5.9 cm. It has built‐in LED illumination, and image acquisition takes approximately 6.4 s. Reflectance spectra are recorded per pixel using a complementary metal‐oxide‐semiconductor (CMOS) image sensor, while hyperspectral data and RGB images are captured simultaneously on two separate sensors.

### Skin Tone Classification

#### Individual Typology Angle (ITA)

Before transforming images to the CIELab colour space, image intensities were scaled using the Value component in the HSV colour space, according to the following formulas:

$$V_{\text{corrected}}=\frac{V-V_{\min}}{1-V_{\min}}$$

$$V_{\text{scaled}}=0.5+V_{\text{corrected}}\times 0.5$$

To indicate the range of skin and mucosa tones of study participants, ITA values were calculated using the equation shown below [32].

$$\mathrm{ITA}=\left[\tan^{-1}\left(\frac{L^{*}-50}{b^{*}}\right)\right]\cdot \frac{180}{\pi }$$

#### Spectral Data and Tissue Indices

These values were calculated according to the following formulas [33]:

$$\mathrm{NIR}=\frac{\text{mean}\left(A_{\left[825\mathrm{nm}-925\mathrm{nm}\right]}\right)}{\text{mean}\left(A_{\left[655\mathrm{nm}-735\mathrm{nm}\right]}\right)}$$

$$\mathrm{THI}=\frac{\text{mean}\left(A_{\left[785\mathrm{nm}-825\mathrm{nm}\right]}\right)}{\text{mean}\left(A_{\left[530\mathrm{nm}-590\mathrm{nm}\right]}\right)}$$

$$\mathrm{TWI}=\frac{\text{mean}\left(A_{\left[955\mathrm{nm}-980\mathrm{nm}\right]}\right)}{\text{mean}\left(A_{\left[880\mathrm{nm}-900\mathrm{nm}\right]}\right)}$$

$$\mathrm{St}O_{2}=\frac{\min\left({A^{\prime \prime }}_{\left[570\mathrm{nm}-590\mathrm{nm}\right]}\right)}{\min\left({A^{\prime \prime }}_{\left[570\mathrm{nm}-590\mathrm{nm}\right]}\right)+\min\left({A^{\prime \prime }}_{\left[740\mathrm{nm}-780\mathrm{nm}\right]}\right)}$$

In these formulas, *A* represents the reflectance spectra.

#### Skin Tone Encoding in Unsupervised and Supervised HSI Clustering

The parameters used in the GMM are described in Table A1.

**TABLE A1:** Values of the parameters in the GMM with a short explanation.

Parameter	Value	Explanation
Number of components	5	Maximum number of clusters that can be made
Maximum iterations	1000	Iterations performed by the model to ensure convergence
Covariance type	Full	Each component gets its own covariance matrix
Shared covariance	False	Each cluster gets its own covariance matrix
Regularisation value	1e−4	Small value to ensure that estimated covariances have positive values
Replicates	10	Performs different fits with different initialisations
Weights	1/number of samples per group	Used because of class imbalance
Start	Plus	Initialises the GMM with a variant of the k‐means++ algorithm

The FCN consisted of an input layer with 100 features, followed by three fully connected layers with 512, 256, and 128 neurons, respectively. After each dense layer, a batch normalisation layer and a ReLU activation function were implemented. Additionally, a dropout layer with a dropout rate of 0.5 was used after the first fully connected layer to decrease overfitting. The last layer consisted of a fully connected layer with 5 output neurons, corresponding to the ITA classes, followed by a softmax activation function and a classification layer. For training the model, an Adam optimizer with a maximum of 60 epochs was used.

## Appendix B

**TABLE B1:** Participant characteristics (*n* = 45).

Participant ID	Age	BMI	Menopause	Mucosal data available	ITA value skin	ITA classification
1	48	26.2	Before	Yes	71.9	Very light
2	46	21.3	Before	Yes	72.7	Very light
3	51	23.9	After	Yes	25.8	Tan
4	55	20.0	After	Yes	21.0	Tan
5	55	45.3	After	Yes	55.9	Very light
6	63	19.3	After	Yes	60.7	Very light
7	57	22.3	After	Yes	51.0	Light
8	43	24.1	Before	Yes	42.3	Light
9	53	25.1	Before	Yes	47.0	Light
10	52	25.9	Before	Yes	52.4	Light
11	64	21.9	After	Yes	66.8	Very light
12	60	23.9	After	Yes	64.0	Very light
13	59	22.0	After	Yes	69.8	Very light
14	59	20.7	After	Yes	75.8	Very light
15	61	33.5	After	Yes	56.1	Very light
16	48	23.1	During	Yes	34.5	Intermediate
17	45	19.5	Before	Yes	65.2	Very light
18	56	31.6	After	No	70.3	Very light
19	64	24.2	After	Yes	57.4	Very light
20	55	25.6	After	Yes	75.5	Very light
21	55	26.4	After	Yes	46.6	Light
22	62	39.3	After	Yes	57.0	Very light
23	56	28.5	After	Yes	65.0	Very light
24	52	20.2	After	Yes	47.2	Light
25	59	21.3	After	No	70.2	Very light
26	53	24.1	After	No	71.6	Very light
27	48	26.4	Before	Yes	39.4	Intermediate
28	46	24.2	Before	No	35.4	Intermediate
29	30	21.2	Before	Yes	71.1	Very light
30	53	30.5	After	Yes	13.8	Tan
31	45	20.9	Before	Yes	45.1	Light
32	53	21.9	During	Yes	20.2	Tan
33	45	23.7	Before	Yes	69.7	Very light
34	40	22.2	Before	Yes	39.7	Intermediate
35	60	24.0	During	No	−0.7	Brown
36	49	32.5	Before	Yes	7.5	Brown
37	41	21.0	Before	Yes	21.1	Tan
38	58	23.7	After	Yes	28.0	Tan
39	45	22.4	Before	Yes	49.7	Light
40	57	22.9	After^a^	Yes	32.0	Intermediate
41	63	24.8	After	Yes	37.7	Intermediate
42	40	21.0	Before	Yes	56.5	Very light
43	57	26.7	Unknown^b^	Yes	45.7	Light
44	51	28.7	Before	Yes	24.3	Tan
45	57	20.2	After	Yes	40.8	Intermediate

^a^ Due to oophorectomy.

^b^ Due to IUD.

## Appendix C

Additional parameters that are used to fully characterise colours are *C*_*ab*_* and *h*_*ab*_. The chroma, *C*_*ab*_*, describes the distance to the neutral grey centre. This parameter is a measure of the saturation and intensity of the colour; it can be calculated according to the following formula [34].

$$C_{\mathrm{ab}}^{*}=\sqrt{a^{*2}+b^{*2}}$$

The Hue angle, *h*_*ab*_, is the angle in the *a**–*b** plane that determines the colour tone and can be calculated by use of the following formula [34].

$$h_{\mathrm{ab}}=\arctan\left(\frac{b^{*}}{a^{*}}\right)$$

In the scatterplot, it can be seen that *C**_*ab*_ is inversely related to *L**, indicating that darker samples had more saturated colours. The *h*_*ab*_ values increase with *L**, indicating that lighter skin colours tend to have higher hue values. As *h*_*ab*_ is derived from the ratio of *b** to *a**, this correlation suggests that the yellow component increases compared to the red component. In contrast, the lower *L** values have a Hue angle closer to red‐yellow, which corresponds to a higher amount of melanin and thus a darker skin. Finally, the positive correlation between *a** and *b** indicates that redness and yellowness increase together.

## Appendix D

In Figure D1, the scatterplots according to the first two principal components are visualised. In the figure, on the left, the actual ITA‐based labels are mapped to the datapoints, and on the right side, the GMM predicted labels are mapped. In this figure, it is clearly visible that the GMM combined the ‘tan’ and ‘brown’ groups, and that the last class created by the GMM is located on the opposite side compared to the true classes in the very light skin tone region.
